# Endogenous–Exogenous Analyses of the Solidification Structure in 475 mm Extra-Thick Slabs: Columnar-to-Equiaxed Positioning and Effect of Strand Electromagnetic Stirring

**DOI:** 10.3390/ma18102179

**Published:** 2025-05-08

**Authors:** Kezai Yu, Lijun Xu, Yanling Zhang, Haibo Zhang, Zhonghua Zhan

**Affiliations:** 1 National Engineering Research Center of Continuous Casting Technology, Central Iron and Steel Research Institute, Beijing 100081, Chinalyzhan1005@163.com (Z.Z.); 2 Material Digital R&D Center, Central Iron and Steel Research Institute, Beijing 100081, China; 3 State Key Laboratory of Advanced Metallurgy, University of Science and Technology Beijing, 30 Xueyuan Road, Beijing 100083, China

**Keywords:** solidification structure, columnar-to-equiaxed transition, CAFE, extra-thick slab, S-EMS

## Abstract

The spatial distribution of equiaxed crystal zones during extra-thick slab solidification exerts a critical influence on the mechanical performance of the final product. This investigation establishes a dual-pathway control framework for solidification structure modulation, differentiating between intrinsic regulation through columnar-to-equiaxed transition (CET) positioning and extrinsic intervention via strand electromagnetic stirring (S-EMS) parameter adjustment. The aim is to improve the internal quality of extra-thick slabs, enabling further investigations into the material properties. To achieve this, a solidification heat transfer model along with a cellular automata–finite element model were developed to characterize the thermal conditions at CET initiation, with experimental validation conducted on a 475 mm extra-thick slab. The systematic analysis identified a significant correlation between continuous casting parameters, alloy concentrations, and CET positioning, while S-EMS experiments further elucidated the distribution patterns of the solidification structure and the formation mechanism of the white band in the mushy zone. This methodology bridges computational metallurgy with process engineering, offering systematic guidance for solidification structure control in extra-thick slabs.

## 1. Introduction

Extra-thick slabs, as essential structural materials for load-bearing components in marine engineering, defense systems, and bridge construction, offer significant economic and environmental benefits due to their superior energy efficiency and reduced carbon footprint [1,2]. As structural demands expand to encompass extended spans, elevated load capacities, and extreme operational conditions—including fire resistance and marine corrosion—Q355 steel, a high-strength, low-alloy structural grade, has become indispensable for modern infrastructure owing to its exceptional strength-to-weight ratio, weldability, and cost-effectiveness [3]. Concurrently, the increasing global emphasis on sustainable manufacturing has driven the demand for high-performance steels produced through eco-friendly processes, necessitating stricter quality standards to meet evolving industrial requirements [4]. However, the continuous casting (CC) of Q355 steel extra-thick slabs remains technically challenging, as extended solidification times and reduced cooling rates often result in severe internal defects, such as centerline segregation and shrinkage porosity [5]. The equiaxed crystal ratio (ECR) within the solidification structure serves as a critical determinant of defect suppression. Crucially, equiaxed grains facilitate a uniform redistribution of the solute-rich residual liquid in the mushy zone through their isotropic morphology, thereby inhibiting solidification bridge formation and mitigating macro-segregation and shrinkage defects [6]. Consequently, optimizing the as-cast structure of extra-thick slabs during CC has become a key priority for advancing sustainable, high-efficiency steel production.

**Figure 1:**
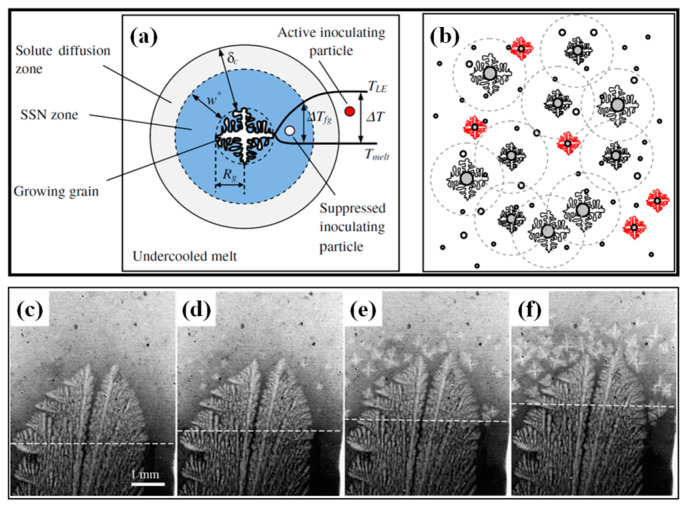
Schematic description of the solute-suppressed nucleation zone (**a**) around an individual crystal and (**b**) developed by an ensemble of growing crystals; (**c**–**f**) a radiograph sequence showing the CET [11].

Contemporary strategies for optimizing solidification structures with enhanced ECR and homogeneity employ dual methodologies, intrinsic regulation of columnar-to-equiaxed transition (CET) initiation and extrinsic control through electromagnetic stirring (EMS), to improve nucleation and growth kinetics [7,8,9]. The CET is positioned as a critical microstructural indicator in extra-thick steel slabs, dictating grain morphology distributions. Hunt’s seminal analytical model [10], incorporating the diffusion coefficient, partition coefficient, and nucleation density, posits that columnar crystal growth ceases via mechanical blocking when the equiaxed crystal volume fraction exceeds 0.49. Similarly, Feng et al. [11] observed through X-ray imaging that mechanical blocking triggers the CET process, where migrating equiaxed grains accumulate at the columnar front, thereby suppressing columnar growth (see Figure 1). Complementarily, Martorano et al. [12] formulated a solute redistribution theory, proposing that the CET initiates when solidification front supercooling is neutralized through solute dissipation during equiaxed grain evolution. While mechanistic interpretations vary, the CET inevitably occurs in CC processes. The CET criteria exhibit composition-dependent variability and are influenced by the alloy chemistry, cooling parameters, and geometric factors, with thermal gradients, growth velocities, and cooling rates at the solidification front serving as critical determinants [13]. Computational modeling advancements have facilitated predictive simulations of solidification patterns. Niu et al. [14] customized the Hunt model for large-diameter blooms (500~600 mm), demonstrating a proportional CET criterion escalation from 5.21 to 6.41 °C·s^1/2^·mm^−3/2^ with increasing dimensions. Employing a cellular automaton–finite difference (CA-FD) model, Luo et al. [15] analyzed competitive growth between equiaxed and columnar crystals in high-carbon steel billets during solidification. Parallel studies by Shibata et al. [16] and Lekakh et al. [17] developed CET criteria for 200 mm-thick conventional slabs and heavy rectangular blooms using stainless steel prototypes, achieving accurate CET localization under controlled cooling. Nevertheless, the current CET models remain primarily validated for conventional sections or blooms, restricting their utility for extra-thick slabs (>400 mm). Operational limitations in dendritic parameter acquisition further impede industrial implementation. Thus, external field modulation techniques like EMS retain crucial importance for solidification structure refinement in extra-thick slabs with substantially increased cross-sections.

EMS technologies have matured as a critical methodology for solidification structure optimization in metallurgical applications [8]. The final grain size serves as a critical determinant of the material’s mechanical performance [18,19]. Strand electromagnetic stirring (S-EMS), a prevalent technique in industrial CC production, achieves microstructural refinement through the controlled enhancement of the ECR [20]. Yao et al. [21] employed a coupled experimental and computational approach to analyze 230 mm-thick slabs, revealing that elevating the S-EMS current from 0 to 150 A modified dendrite evolution patterns, thereby increasing the ECR from 13.04% to 37.52% and mitigating centerline segregation. A parallel scientific article by Wu et al. [22] on analogous slabs confirmed progressive ECR improvements at the slab center with higher stirring frequencies and currents. Despite these advances, asymmetric solidification patterns persist as a critical challenge in bow-type continuous casters. Xiao et al. [23] investigated a 230 mm-thick slab produced via a curved continuous caster, observing that while S-EMS elevated ECR from 8% to 33%, it concurrently induced heterogeneous grain structures characterized by a pronounced shift of the equiaxed crystal zone toward the fixed side. Hu et al. [24] developed a 2D thermal-fluid model for φ300 mm 42CrMoA steel round blooms incorporating mold EMS, S-EMS, and final EMS, while accounting for gravitational effects on grain nucleation and transport. Their simulations predicted loose-side and fixed-side structural asymmetries with a 5.5% deviation in columnar zone length quantification. Wang et al. [25] established a multiscale cellular automaton framework for 160 × 160 mm^2^ billets, identifying free equiaxed crystal sedimentation from the loose side to fixed-side columnar fronts as the primary asymmetry mechanism. Although grain dynamics and ECR enhancement in blooms and billets have been thoroughly characterized [26,27,28], spatial heterogeneity mechanisms in large cross-sectional slabs remain poorly resolved. This knowledge gap is particularly acute for 475 mm extra-thick slabs produced via vertical-curved continuous casting—the maximum thickness achievable in this industrial configuration.

This study establishes a validated solidification heat transfer model and a cellular automaton–finite element model for a 475 mm ultra-thick slab. The solidification structure control strategy was systematically decomposed into dual regulatory dimensions: endogenous optimization through CET positioning and extrinsic modulation via S-EMS parameter engineering. Initially, a streamlined CET criterion was formulated specifically for ultra-thick slabs through a thermodynamic analysis of the phase transition initiation conditions, establishing quantitative relationships between CET localization and continuous casting parameters/alloy compositions to create a multi-variable optimization matrix. Subsequently, industrial-scale S-EMS trials were executed to evaluate the microstructural characteristics and internal quality metrics across operational parameters, with a particular emphasis on resolving asymmetric solidification patterns. This methodology provides an implementable framework for industrial solidification structure optimization, establishing systematic connections between computational metallurgy predictions and process engineering applications.

## 2. Methodology Descriptions

### 2.1. Heat Transfer and Solidification Model

To balance computational efficiency and accuracy in modeling the dynamic and complex CC process, the following assumptions were applied: heat transfer in the secondary cooling zone was represented using an integrated heat transfer coefficient for slab surface interactions; heat transfer along the casting direction was neglected; and dendrite settling and solid-phase motion during solidification were excluded [29]. A finite element method based slice-moving model was employed to simulate the extra-thick slab CC process. The governing heat transfer equations for the slab are:

(1) $$\frac{\partial }{\partial x}\left(\lambda \frac{\partial T}{\partial x}\right)+\frac{\partial }{\partial y}\left(\lambda \frac{\partial T}{\partial y}\right)=\rho \frac{\partial H}{\partial t}$$

(2) $$H=\int_{0}^{T}C_{p}dT+L_{P}\left(1-f_{S}\right)$$

The initial and boundary conditions in the calculation are given below.

(1) Initial condition

(3) $$T_{0}=T_{1}=1805K$$

(2) Boundary condition

The extra-thick slab passes through the mold, secondary cooling zone, and air cooling zone during the CC process. In the mold stage, heat is dissipated via circulating cooling water in the copper plate. This heat transfer is quantified using instantaneous heat flux density [30], and calculated as follows:

(4) $$q_{m}=\;\left(2.688-B\sqrt{t}\right)\times 10^{3}$$

(5) $$B=\frac{3}{2\sqrt{l/v}}\left(2.688\times 10^{3}-q_{a}\right)$$

Heat transfer in the foot roll section, the remaining secondary cooling zone, and the air cooling zone is governed by water spray cooling, aero-mist cooling, and radiant heat transfer, respectively. The corresponding boundary conditions and parameters are provided in Table 1.

### 2.2. Grain Nucleation Model

In this paper, a continuous non-uniform nucleus model based on a normal distribution is adopted to simulate the solidification structure [32,33]:

(6) $$n\left(\Delta T\right)=\int_{0}^{\Delta T}\frac{dn}{d\left(\Delta T\right)}d\left(\Delta T\right)$$

(7) $$\frac{dn}{d\left(\Delta T\right)}=\frac{n_{\max}}{\sqrt{2\pi }\Delta T_{\sigma }}\exp\left[-\frac{1}{2}{\left(\frac{\Delta T-\Delta T_{n}}{\Delta T_{\sigma }}\right)}^{2}\right]$$

The nucleation parameters and growth parameters are described in Table 2.

### 2.3. Dendrite Tip Growth Kinetics Model

To streamline computational efficiency, dendrite tip growth kinetics are calculated using the Kurz–Giovanola–Trivedi (KGT) model [34,35], which is expressed as follows:

(8) $$v=\alpha_{2}\Delta T^{2}+\alpha_{3}\Delta T^{3}$$

(9) $$\alpha_{2}=[\frac{-\rho }{2mc_{0}{\left(1-k\right)}^{2}\Gamma k}+\frac{1}{mc_{0}(1-k)D}]\frac{D^{2}}{\pi^{2}\Gamma }$$

(10) $$\alpha_{3}=\frac{D}{\pi \Gamma }\cdot \frac{1}{{\left(mc_{0}\right)}^{2}(1-k)}$$

where *α*_2_ and *α*_3_ are the fitted polynomial coefficients of the dendrite tips’ kinetic parameters related to the alloy composition, and the units are m·s^−1^·K^−2^, and m·s^−1^·K^−3^, respectively. The Q355 high-strength structural steel is divided into eight binary systems of Fe-C, Fe-S, Fe-Mn, Fe-Si, Fe-P, Fe-Cr, Fe-Al, and Fe-Ti. The kinetic parameters of dendrite growth in CAFÉ model are shown in Table 3 [36,37].

## 3. Experimental Conditions and Model Verification

### 3.1. Thermo-Physical Properties of Steel

To improve computational efficiency, the thin-slice method was applied to simulate the slab solidification process. A symmetric half-section of the slab was selected for analysis. A geometric model (1000 mm × 475 mm × 10 mm) was constructed in SolidWorks (version 2018), followed by hexahedral mesh generation in ProCAST (version 13.5), yielding a total mesh count of 608,875. The geometric configuration and meshing strategy align with a previous paper [38].

**Figure 2:**
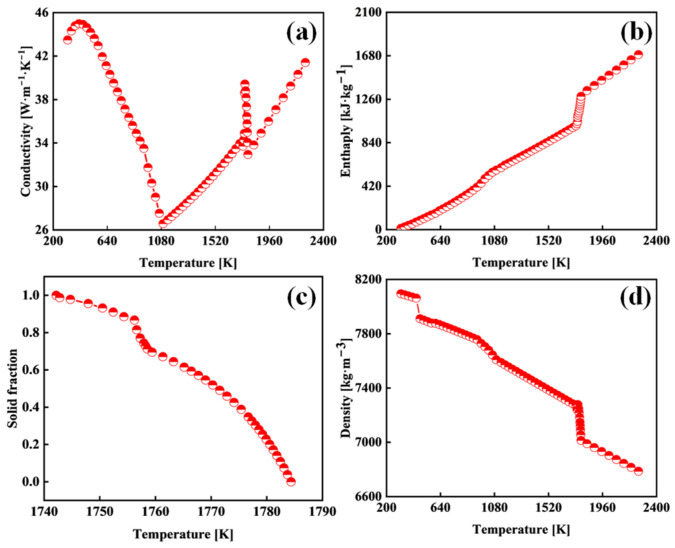
Thermophysical properties of Q355 steel: (**a**) conductivity; (**b**) enthalpy; (**c**) solid fraction; and (**d**) density.

The extra-thick slabs were produced using a vertical curved continuous caster with a machine radius of 12.10 m. The study focused on Q355 low-alloy structural steel, with a composition of 0.163 wt.% C, 0.286 wt.% Si, 1.525 wt.% Mn, 0.021 wt.% P, 0.0042 wt.% S, 0.044 wt.% Al, 0.021 wt.% Cr, and 0.016 wt.% Ti. As shown in Figure 2, this composition was input into ProCAST’s material database to calculate the thermophysical parameters, and the liquidus and solidus temperatures were determined as 1784 K and 1742 K, respectively. The thermal conductivity initially decreased and then increased with decreasing temperature, exhibiting inflection points near 1784 K and 1094 K (Figure 2a) due to the solid–liquid phase transition and hyper-eutectoid transformation, respectively. Enthalpy decreased monotonically with temperature (Figure 2b), while the solid fraction and density exhibited increasing trends (Figure 2c,d). The numerical model incorporated the temperature-dependent material properties of the steel grade outlined in Figure 2.

### 3.2. Verification of the Mathematical Models

#### 3.2.1. Verification of the Solidification Heat Transfer Model

**Figure 3:**
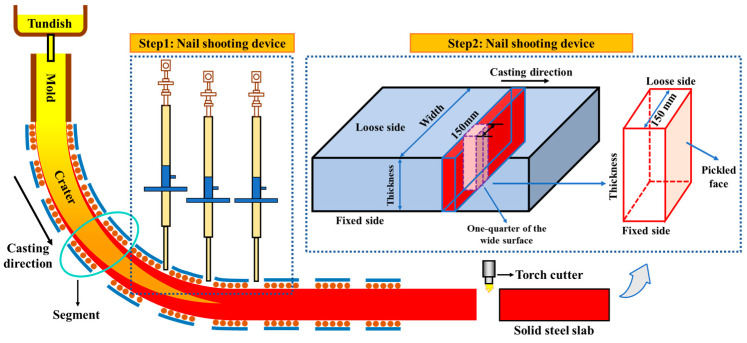
Shot nail sampling schematic diagram.

**Figure 4:**
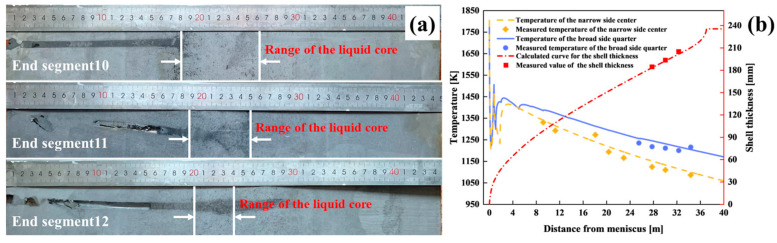
The calculated and the measured results: (**a**) the nail-shooting specimen morphology and (**b**) comparison between the calculated and measured results.

The accuracy of the solidification heat transfer model for 475 mm extra-thick slabs was validated through nail penetration experiments and surface temperature measurements. Nail sampling devices (nail composition: 60Si2Mn) were installed at 1/4 of the slab’s broadside, positioned 27.76 m, 30.03 m, and 32.31 m below the meniscus (corresponding to the ends of sectors 10, 11, and 12 of the caster). Figure 3 illustrates the experimental setup. The post-solidification analysis of the nails assessed the liquid core width and shell thickness based on sulfide diffusion patterns and morphological boundaries (Figure 4a). As shown in Figure 4b, the simulated shell thickness exhibited a maximum deviation of 3.3 mm from the measured values in sectors 10~12, with a relative error (defined as the ratio of the simulated–actual difference to the actual value) below 2%. Infrared thermometry further validated the model at one-quarter broadside and narrow side centerline locations, with temperature differences ≤33 K and errors <2.59%. The close agreement between simulations and measurements confirms the model’s reliability for predicting the solidification behavior in 475 mm slabs.

#### 3.2.2. Validation of the Solidification Structure Model

**Figure 5:**
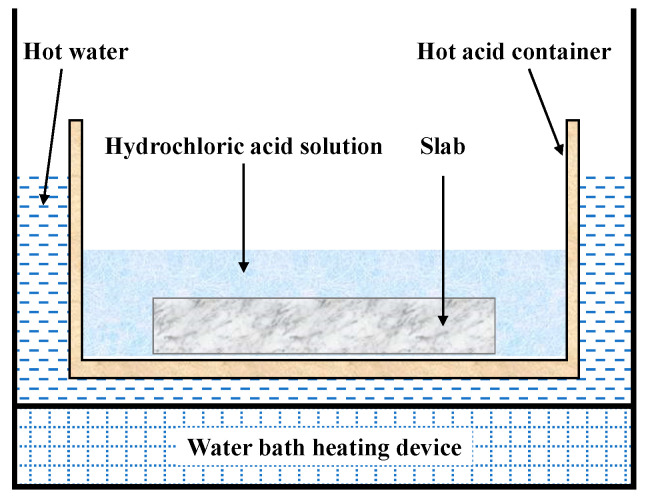
Slab acid etching schematic diagram.

**Figure 6:**
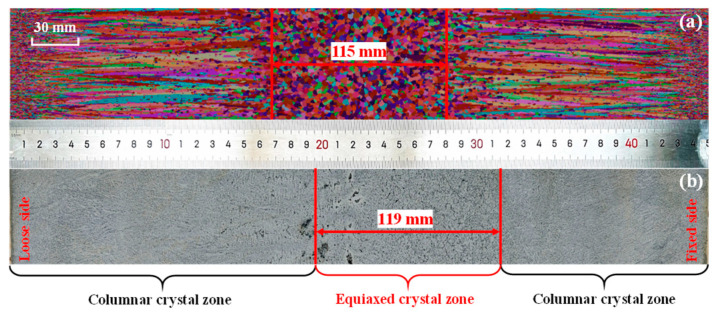
Comparison of the morphology of macro-structure: (**a**) simulated results and (**b**) experimental results.

The post-processed extra-thick slab specimens were etched in a 1:1 (volume ratio) hydrochloric acid (HCl) aqueous solution at a controlled temperature of 343–353 K, as illustrated in Figure 5. Figure 6 compares the solidification structure predicted by the CAFE model with the macroscopic morphology observed in acid-etched specimens. As shown in Figure 6b, the fixed side exhibits restricted columnar crystal growth due to gravity-settled nuclei, leading to divergent structural evolution between the loose and fixed sides. This asymmetry shifts the equiaxed crystal zone toward the fixed side, yielding a measured ECR of 18.44%. The CAFE model predicts a comparable ECR of 19.11%, with a minor discrepancy of 0.67% between the simulated and experimental results. This close alignment confirms the model’s capability to reliably predict solidification structures in extra-thick slabs.

## 4. Results and Discussion

### 4.1. Columnar-to-Equiaxed Crystal Transition Analysis

#### 4.1.1. Temperature Conditions at the CET Initiation Position

**Figure 7:**
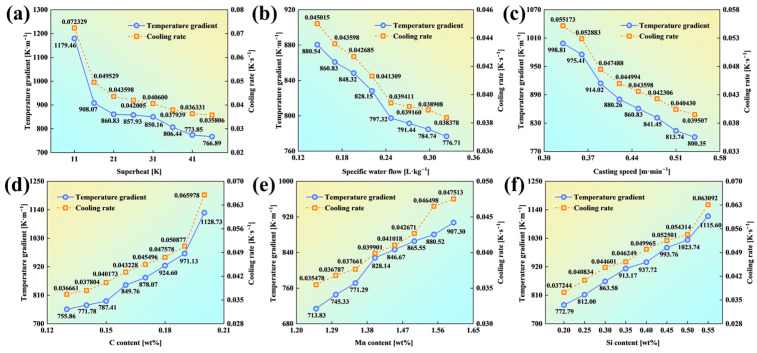
*G* and *L* at the CET initiation position under varying conditions: (**a**) superheat; (**b**) specific water flow; (**c**) casting speed; (**d**) C content; (**e**) Mn content; and (**f**) Si content.

The temperature conditions at the CET initiation position were analyzed using the validated solidification heat transfer and CAFE models, with variations in the steel composition and CC parameters. Following the standard for the solidification front analysis in metals [7], the temperature gradient (*G*) and cooling rate (*L*) at a solid fraction of 0.3 (*f_S_* = 0.3) were extracted via the control variable method (Figure 7). Key observations include (1) process parameters, where both *G* and *L* at the CET initiation position decrease with higher superheat, increased secondary cooling intensity (specific water flow), or faster casting speeds; and (2) alloying elements, as *G* and *L* rise with elevated carbon (C), manganese (Mn), and silicon (Si) contents. To quantify the relative impacts of CC parameters and the elemental composition on *G* and *L*, an influence rate (*λ*) is defined as follows:

(11) $$\lambda =\frac{M_{\max}-M_{\min}}{M_{\min}}\times 100\%$$

**Figure 8:**
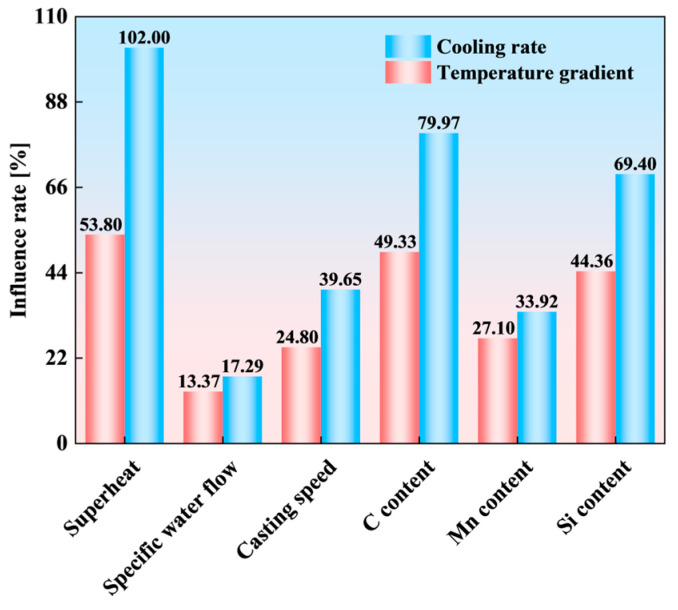
Sensitivity of G and L at the CET initiation position to different variables.

As indicated in Figure 8, superheat, the C content, and Si content exhibit the strongest influences on *G* and *L*. At CET initiation, superheat, C, and Si account for 102%, 79.97%, and 69.40% of the observed influence rates on *G*, respectively. Their influence rates on *L* are similarly dominant, contributing 53.80%, 49.33%, and 44.36%, respectively. These parameters significantly outweigh other thermal factors in their impacts on the CET position. These findings underscore the necessity of accounting for the alloy composition when evaluating the thermal conditions at CET initiation.

In melt solidification, a stable fully equiaxed structure emerges when the volume fraction of equiaxed crystals at the columnar front exceeds a critical threshold. Hunt et al. [10] proposed that CET occurs when this fraction surpasses 0.49. However, the Hunt model’s complexity in determining required parameters limits its practical application. To address this, Kurz et al. [39] introduced a simplified criterion by assuming negligible heterogeneous nucleation undercooling (Δ*T_N_* ≈ 0) due to rapid dendrite tip growth rates. This yields the following form:

(12) $$G<0.617\cdot {N_{0}}^{1/3}\cdot \left[1-{\left(\Delta T_{N}/\Delta T_{C}\right)}^{3}\right]\cdot \Delta T_{C}$$

(13) $$\Delta T_{C}=\Delta T_{0}{\left(a_{1}\cdot V\right)}^{1/n}$$

**Figure 9:**
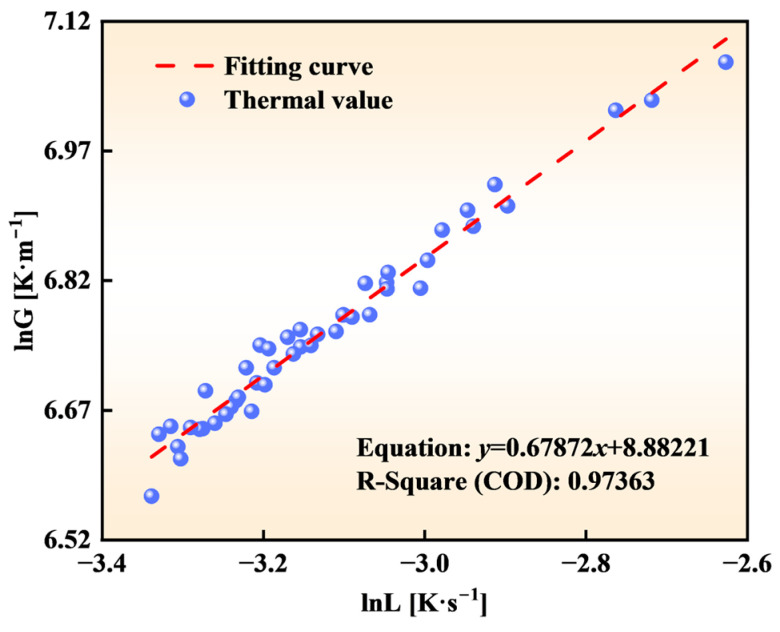
ln*G* and ln*L* fitting curve.

Given that the ratio Δ*T_N_*/Δ*T_C_* ≪ 1, Equation (12) simplifies to *G^n^*/*V* < *B*, where *B* and *n* are constants dependent on the alloy system [17]. Under steady-state conditions, *L*, *G* and *V* are related by *L* = *G* × *V*. Substituting this relationship into the simplified criterion yields the CET condition:

(14) $$\frac{G^{m_{1}}}{L}<E$$

where *m*_1_ and *E* are material-dependent constants. Equation (14) indicates that the CET initiates when the ratio $G^{m_{1}}/L$ falls below the critical threshold *E*. This criterion can be expressed as follows:

(15) $$m_{1}\ln G=\ln L+\ln E\Rightarrow \ln G=\frac{1}{m_{1}}\ln L+\frac{1}{m_{1}}\ln E\Rightarrow y=\frac{1}{m_{1}}x+e$$

where *y* = ln*G*; *x* = ln*L*; and *e* = ln*E*/*m*_1_. Using the *G* and *L* values at the CET initiation position across varying conditions, Equation (15) was fitted to the data (Figure 9). The fit demonstrated excellent agreement (*R*^2^ = 0.97363), yielding parameters *a*_1_ = 1.47 and *E* = 482,486.0. This approach effectively circumvents the inaccuracies associated with an arbitrary nucleation site density (*N*_0_) selection. For the studied steel grade, the critical temperature conditions at the CET initiation position are

(16) $$\frac{G^{1.47}}{L}<482486.07$$

A comparison between the simulated CET conditions and experimental parameters showed that the discrepancies remained below 8.52%. These results confirm that Equation (16) serves as a validated criterion for CET position determination in the studied steel grades.

#### 4.1.2. Relationship Between the CET Initiation Position and Parameters

**Figure 10:**
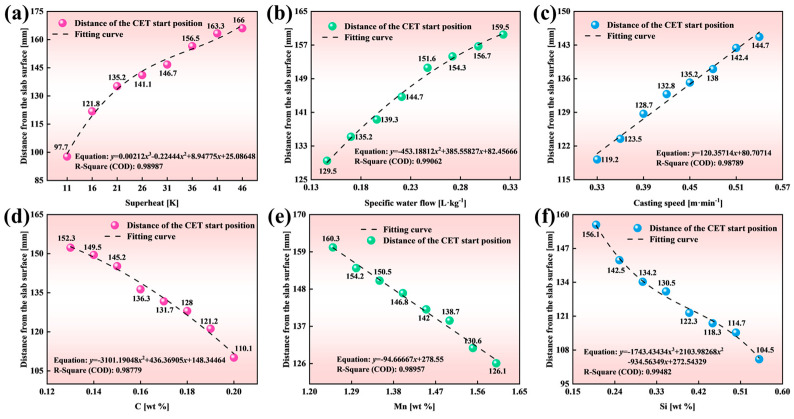
Fitting curves for different parameters: (**a**) superheat; (**b**) specific water flow; (**c**) casting speed; (**d**) C content; (**e**) Mn content; and (**f**) Si content.

Figure 10 shows the depth of the CET initiation position beneath the slab surface, as predicted by the heat transfer and solidification structure models under varying parameters: superheat (11 K~46 K), specific water flow (0.146 L kg^−1^~0.323 L kg^−1^), and casting speed (0.33 m·min^−1^~0.54 m·min^−1^).

As shown in Figure 10a–c, the distance from the slab surface to the CET initiation position increases with superheat, specific water flow, and casting speed. Firstly, the relationship between superheat (*x*_1_) and CET depth (*y*_1_) is quantified by Equation (17), which achieves a high coefficient of determination (*R*^2^ = 0.98987). Increasing superheat extends the CET depth, though the rate of increase diminishes at a higher superheat. This occurs because an elevated superheat promotes the melting of free equiaxed crystals in the molten steel, reducing the number of viable nuclei at the columnar crystal front. Consequently, equiaxed crystal nucleation and growth are suppressed, delaying CET behavior. Secondly, Equation (18) quantifies the relationship between the CET depth (*y*_2_) and specific water flow (*x*_2_). The high coefficient of determination (*R*^2^ = 0.99062) confirms that the fitted curve reliably predicts CET positions across the tested range of specific water flow values. Increased specific water flow intensifies supercooling at the columnar front, favoring sustained columnar crystal growth. This delays CET occurrence and extends the distance of the CET initiation position from the slab surface. Next, the relationship between the casting speed and the depth of the CET initiation position is quantified in Equation (19), achieving a coefficient of determination *R*^2^ = 0.99216.

(17) $$y_{1}=0.00212{x_{1}}^{3}-0.22444{x_{1}}^{2}+8.94775x_{1}+25.08648(11\leq x_{1}\leq 46)$$

(18) $$y_{2}=-453.18812{x_{2}}^{2}+385.55827x_{2}+82.45666(0.146\leq x_{2}\leq 0.323)$$

(19) $$y_{3}=120.35714x_{3}+80.70714(0.33\leq x_{3}\leq 0.54)$$

As illustrated in Figure 10d–f, the influence of alloy compositions on the CET initiation position was analyzed by statistically correlating its depth beneath the slab surface with variations in carbon (C: 0.13~0.20 wt.%), manganese (Mn: 1.25~1.60 wt.%), and silicon (Si: 0.20~0.55 wt.%) contents.

**Figure 11:**
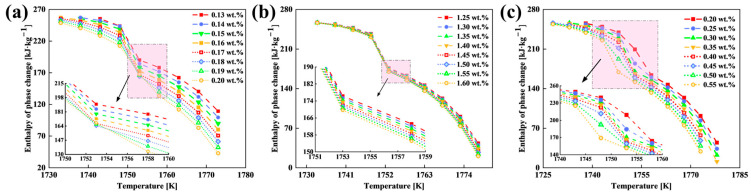
Enthalpy of the phase change with different elemental contents: (**a**) C content; (**b**) Mn content; and (**c**) Si content.

The relationship between the carbon content (*x*_4_) and CET depth (*y*_4_) is modeled by Equation (20) (*R*^2^ = 0.98779), revealing a strong negative correlation. This inverse relationship stems from two aspects. From a kinetic perspective, lower liquidus temperatures (Table 4) enhance grain nucleation kinetics, increasing the nucleus density and accelerating CET initiation [40]. Concurrently, the reduced dendrite tip growth rate (parameter *α*_3_) suppresses columnar crystal growth, promoting competitive growth between equiaxed and columnar structures [41]. From a thermophysical perspective, a higher carbon content decreases the alloy’s phase change enthalpy (Figure 11a), shortening the crystallization time at the solidification front. This restricts grain growth while favoring nucleation, further facilitating the CET [42]. Moreover, Equation (21) describes the relationship between the manganese content (*x*_5_) and CET depth (*y*_5_) (*R*^2^ = 0.98957), with elevated Mn contents similarly reducing the CET depth. This trend is primarily driven by Mn’s suppression of columnar crystal expansion through a reduction in the dendrite tip growth kinetic parameter (*α*_3_) (Table 4). Although Mn marginally affects phase change enthalpy and solidus temperatures (Figure 11c), its limited influence on thermophysical properties minimizes disruptions to solidification dynamics and structural uniformity in the extra-thick slab. Finally, Equation (22) quantifies the relationship between the silicon content (*x*_6_) and CET depth (*y*_6_) (*R*^2^ = 0.99482), demonstrating that higher Si contents accelerate CET initiation. Silicon lowers the liquidus temperature (Table 4), enhancing nucleation kinetics and viable nuclei density. Reduced dendrite tip growth rates (*α*_3_) further suppress columnar crystal expansion. Additionally, the increased Si content significantly decreases phase change enthalpy (Figure 11c), shortening the crystallization time at the solidification front. This dual effect restricts grain growth while promoting nucleation, thereby accelerating the CET.

(20) $$y_{4}=-3101.19048{x_{4}}^{2}+436.36905x_{4}+148.34464(0.13\leq x_{4}\leq 0.20)$$

(21) $$y_{5}=-94.66667x_{5}+278.55(1.25\leq x_{5}\leq 1.60)$$

(22) $$\begin{array}{c} y_{6}=-1743.43434{x_{6}}^{3}+2103.98268{x_{6}}^{2}-934.56349x_{6}\\ +272.54329(0.2\leq x_{6}\leq 0.55) \end{array}$$

To enable practical application in industrial settings, a comprehensive predictive model for the CET initiation position was developed by integrating key CC parameters and alloying elements, i.e., *y* = *a·y*_1_ + *b·y*_2_ + *c·y*_3_ + *d·y*_4_ + *e·y*_5_ + *f·y*_6_ + *g*. The CET depth is expressed as follows:

(23) $$\begin{array}{c} y=0.96819y_{1}+0.98189y_{2}+1.67767y_{3}+0.97364y_{4}\\ +0.98738y_{5}+0.96392y_{6}-678.9288 \end{array}$$

Table 5 summarizes the fitted standard errors and confidence intervals. The model achieves a coefficient of determination *R*^2^ = 0.98670, demonstrating its capability to predict CET positions across variations in CC parameters and alloying elements (C, Si, and Mn). This integrated framework combines Equations (17)–(22) into Equation (23), enabling a systematic evaluation of CET behavior under diverse industrial conditions.

(24) $$\begin{array}{l} y=0.00205x_{1}^{3}-0.21730x_{1}^{2}+8.66312x_{1}-444.98088x_{2}^{2}+378.57581x_{2}\\ +201.91956x_{3}-3019.44310x_{4}^{2}+424.86636x_{4}-93.47198x_{5}\\ -1680.53123x_{6}^{3}+2028.07098x_{6}^{2}-900.84444x_{6}+922.83070\\ (11\leq x_{1}\leq 46,0.146\leq x_{2}\leq 0.323,0.33\leq x_{3}\leq 0.54,\\ 0.13\leq x_{4}\leq 0.20,1.25\leq x_{5}\leq 1.60,0.2\leq x_{6}\leq 0.55) \end{array}$$

### 4.2. Analysis of the Effects of S-EMS on the Internal Structure and Quality of the Extra-Thick Slab

#### 4.2.1. Analysis of the Effect S-EMS on Internal Quality

This paper examines the impact of S-EMS on the internal quality and solidification structure of 475 mm extra-thick slabs through industrial trials. With the S-EMS position fixed, the parameters were optimized under stable production conditions to enhance slab quality. Building on the prior computational analysis, the trials were designed, as outlined in Table 6.

**Figure 12:**
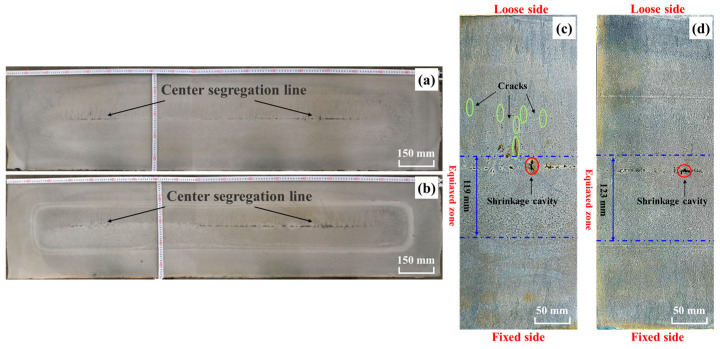
Low-magnification images of slabs with and without S-EMS: (**a**) cross-section of slabs under scheme 1; (**b**) cross-section of slabs under scheme 2; (**c**) zone at one-quarter of the broadside under scheme 1; and (**d**) zone at one-quarter of the broadside under scheme 2.

Figure 12a,b compare cross-sectional macrostructures of slabs processed with and without S-EMS after acid etching (Schemes 1 and 2, respectively). Both conditions exhibit a distinct black segregation line in the central zone. To further analyze internal defects, a specimen from the one-quarter broadside position was hot-acid-etched (Figure 12c,d). Without S-EMS, the slab exhibits a center ECR of 26.44%, along with multiple shrinkage cavities in the central region and cracks on the loose side, classified as severity rank 1.5 under the YB/T 4003-2016 standard [43]. Implementing S-EMS increased the grain density, refined dendritic structures, and elevated the ECR to 27.33%. Concurrently, the maximum central shrinkage area decreased by 24.7% (from 57.94 mm^2^ to 43.58 mm^2^). However, as shown in Figure 12a,b, the slower solidification at 0.45 m·min^−1^ casting speed results in an inadequate shell thickness to counteract the molten steel hydrostatic pressure, causing pronounced bulging on the narrow sides. This defect likely diminishes the efficacy of S-EMS in improving internal quality.

**Figure 13:**
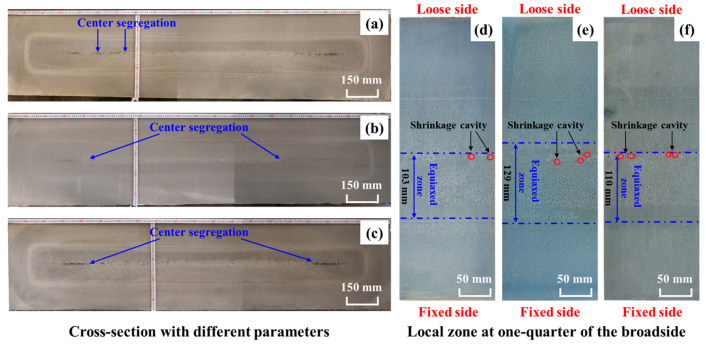
Low-magnification images of cross-sections with different parameters: (**a**,**d**) scheme 3; (**b**,**e**) scheme 4; and (**c**,**f**) scheme 5.

Our prior study [38] demonstrated that the casting speed exerts a more significant influence on the solidification of extra-thick slabs than superheat or specific water flow, with lower speeds enhancing the solidification process. To optimize this process while balancing the steel plant’s production output and operational constraints, the casting speed was reduced to 0.42 m·min^−1^. The post-adjustment analysis (Figure 13d) revealed substantial improvements in slab quality: the ECR reached 22.89% and the maximum central shrinkage area decreased to 13.48 mm^2^, outperforming the results achieved at the higher casting speed of 0.45 m·min^−1^. However, the cross-sectional analysis under constant S-EMS parameters at 0.42 m·min^−1^ (Figure 13a) indicates persistent macroscopic segregation in the slab center, accompanied by Class C 1.5 central porosity and minor intermediate cracks (severity rank: 0.5). These defects likely arise from the trade-offs inherent to reduced casting speeds. While slower solidification increases the shell thickness—mitigating bulging deformation and partially alleviating center segregation and porosity—it concurrently extends the thermal gradient duration, exacerbating residual segregation and cavity formation.

An analysis of Figure 13a,b,d,e reveals that reducing the S-EMS frequency from 5 Hz to 3.5 Hz at a constant current intensity of 570 A significantly enhances the slab quality. The ECR increases from 22.89% to 28.67%, while the maximum shrinkage area decreases by 37% (13.48 mm^2^ to 8.50 mm^2^). Concurrently, the central porosity severity declines from Class 1 to 0.5, and central segregation improves from Class C1.5 to C1.0. These effects arise from the interplay between the stirring intensity and penetration depth in the S-EMS system. Stirring intensity, which is proportional to both current and the square root of frequency, increases with higher frequencies at a fixed current. However, increasing the frequency reduces magnetic flux penetration into the molten steel, confining stirring forces to the slab surface and limiting structural refinement in deeper regions [44]. Thus, the efficacy of S-EMS depends on balancing these interdependent parameters—stirring intensity governs energy input, while penetration depth determines its spatial influence. Together, they modulate solidification dynamics and microstructure formation through mechanisms that remain incompletely resolved [45,46].

As depicted in Figure 13e,f, increasing the current from 420 A to 570 A at a fixed stirring frequency of 3.5 Hz increased the stirring intensity. This suppressed columnar crystal growth while promoting nucleation, thereby elevating the ECR from 24.44% to 28.67%. Under national standard ratings, slabs produced at 570 A exhibited superior internal quality compared to those at 420 A: central porosity decreased from Class 1.0 to 0.5, and the total length of central segregation zones shortened from approximately 300 mm to 200 mm. Furthermore, segregation bands in the 420 A specimens were broader, as evidenced in Figure 13b,c.

A distinct white band is observed within the black rectangle in Figure 13d–f, located approximately 125~130 mm from the slab surface. Under S-EMS, such bands commonly form due to a reduced solute element concentration in the affected region, which promotes negative segregation. The lower solute content enhances corrosion resistance in this area, yielding a lighter appearance after acid etching. This phenomenon occurs because molten steel within the crater begins to flow turbulently under electromagnetic forces as the slab enters the S-EMS region. The resulting fluid motion disperses the inter-dendritic solute concentration at the solidification front, thereby reducing the localized solute content and forming the bright white band [47,48].

**Figure 14:**
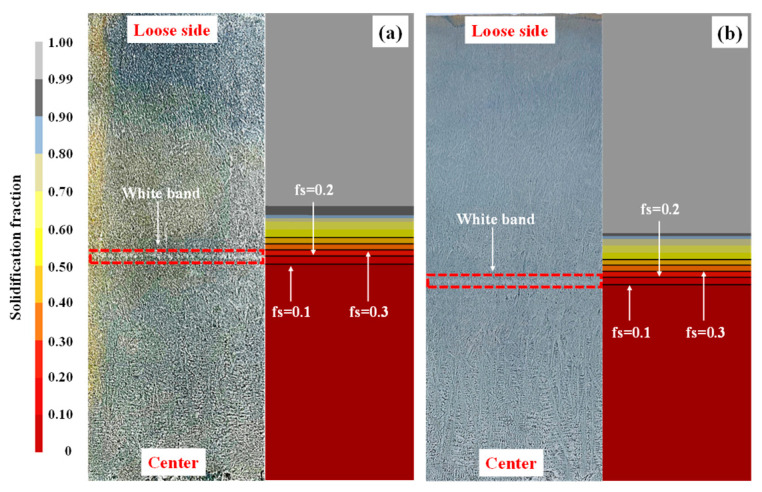
White bright band actual location vs. predicted location: (**a**) 0.45 m·min^−1^ and (**b**) 0.42 m·min^−1^.

Figure 14 compares the positions of white bands and predicted solid fraction (*f_s_*) profiles at casting speeds of 0.42 m·min^−1^ and 0.45 m·min^−1^. TAKAHASHI et al. [49] categorized the mushy zone into three regions based on molten steel flow: *q*_1_ (0.7 > *f_s_* > 0.3), inter-dendritic flow, where molten steel permeates between dendritic structures; *q*_2_ (*f_s_* < 0.3), the liquid-phase-dominated region, which is susceptible to solute washout; and *p* (*f_s_* > 0.7), immobile region, where solidified structures encapsulate residual liquid. Combining the white band formation mechanism, mushy zone delineation, and positional comparisons across casting speeds, this study demonstrates that white bands predominantly form in mushy zone regions where *f_s_* < 0.3. This aligns with prior observations by Xia et al. [50], confirming that solute depletion in liquid-phase-dominated regions (*q*_2_) drives white band formation.

#### 4.2.2. Analysis of the Effect of S-EMS on the Solidification Structure

As shown in Figure 13, a white band appears within the dashed black frame on the slab’s loose side. Under S-EMS, the solidification structure near this band exhibits a greater density, flanked by columnar crystal zones on both sides. This morphology arises from an insufficient electromagnetic stirring force, which fails to generate adequate liquid flow to fully suppress columnar crystal growth. When the slab exits the S-EMS region, the gravitational settling of equiaxed crystals on the loose side is insufficient to mechanically block grain growth. Consequently, grains continue to align with the thermal gradient, forming secondary columnar crystals. Figure 13 further reveals an asymmetric solidification structure in the extra-thick slab: the equiaxed crystal zone is predominantly located near the fixed side and exhibits higher compactness compared to the loose side.

**Figure 15:**
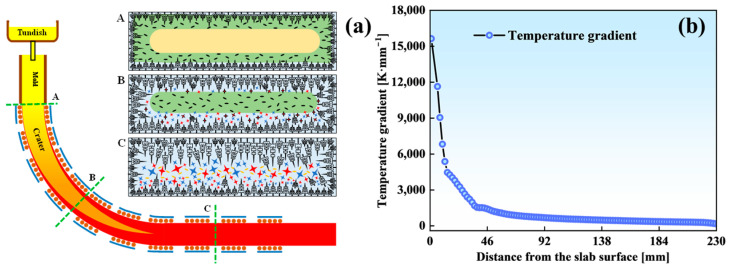
Solidification process and temperature gradient at the solidification front of the extra-thick slab: (**a**) the formation process of solidification structure characteristics; (**b**) the temperature gradient of the solidification front at 0.42 m·min^−1^.

Figure 15 illustrates the asymmetric distribution of the central equiaxed crystal zone and the evolution of solidification structures in the extra-thick slab. Positions A, B, and C correspond to three distinct stages of solidification during CC: mold exit, secondary cooling zone, and final slab solidification, respectively. Stage A (mold exit): As molten steel flows from the tundish into the mold, nozzle-induced horizontal flow generates turbulence. This turbulent flow erodes the solidification interface, fracturing or fusing dendritic arms [51]. Some free dendrites remelt to reduce the superheat, while others remain suspended in the flowing steel (Figure 15a, stage A). Stage B (secondary cooling zone): Upon entering the secondary cooling zone, the superheat in the molten crater dissipates via shell heat transfer and crystal nucleus melting. In the S-EMS region, electromagnetic forces drive fractured dendritic fragments from the solidification front into the crater, supplying additional nucleation sites [21,52]. After exiting the S-EMS region, nuclei settle on the fixed side under gravity in the caster’s curved section, mechanically blocking columnar crystal growth and accelerating the CET. Conversely, insufficient grain accumulation at the loose side’s solidification front prevents secondary columnar crystal formation. Simultaneously, superheated steel exchanges heat with free crystals; larger crystals either melt or exhibit limited growth, preventing equiaxed crystal coarsening near the fixed side (Figure 15a, stage B). Stage C (final solidification): Figure 15b shows the temperature gradient trend at the solidification front. A reduced gradient near the slab center at solidification completion induces moderate compositional undercooling. With low superheat, existing nuclei grow rapidly under these conditions, promoting coarse equiaxed crystal formation near the slab center. Vacuum suction intensifies concentrated molten steel flow through coarse inter-dendritic channels, ultimately forming localized segregation regions of increased size and density (Figure 15a, stage C).

## 5. Conclusions

(1) A validated solidification heat transfer model and a CAFE model were developed to predict the solidification structure in a 475 mm extra-thick slab. The thermal analysis identified superheat, the C content, and the Si content as the primary factors controlling CET initiation. These variables exhibited the strongest influences on *G*, with relative contributions of 102%, 79.97%, and 69.40%, respectively. Their effects on *L* were similarly significant (53.80%, 49.33%, and 44.36%), far exceeding those of other process parameters. The findings establish an operational framework for strategically modulating superheat, the C content, and the Si content during industrial implementation to optimize solidification microstructure characteristics in extra-thick slab production.

(2) A simplified criterion for the CET in the studied steel grades was derived as *G*^1.47^*/L* < 482,486.07. This criterion, based on the thermal field analysis of extra-thick slabs, mitigates uncertainties in selecting the nucleation density. Furthermore, the CET initiation position was systematically evaluated with varying CC parameters and alloying element concentrations. The developed multi-factor regression model enables precise ECR control in CC operations, providing metallurgists with a predictive framework for the solidification structure.

(3) The integrated analysis of white band localization, predicted solid fraction distributions, and mushy zone formation mechanisms demonstrates preferential white band formation at solidification fronts with *f_S_* < 0.3. Industrial validation trials at a casting speed of 0.42 m·min^−1^ confirmed that optimizing S-EMS parameters to 570 A and 3.5 Hz substantially improved metallurgical quality, yielding a 28.67% maximum ECR at the slab center while reducing the shrinkage porosity to 8.50 mm^2^.

(4) During the solidification of a 475 mm extra-thick slab in a vertical-curved continuous caster, equiaxed grains on the loose side detach from the columnar crystal tips under gravity. These grains either remelt in the high-temperature molten core or settle at the columnar crystal front on the fixed side. The settled grains mechanically obstruct columnar crystal growth on the fixed side, accelerating the CET. Conversely, on the loose side, the insufficient accumulation of free grains at the solidification front fails to sustain secondary columnar crystal growth. This mechanism clarifies that the asymmetric solidification structure in extra-thick slabs originates primarily from free grain sedimentation, challenging the conventional attribution to suboptimal casting parameters.

## Figures and Tables

**Table 1:** Related parameters and boundary conditions of the secondary cooling zone.

Cooling Zone		Length [m]	Water Flow Rate [L·min^−1^]	Computational Formula
Secondary Cooling Zone	Foot roll section (N)	1.82	78.20	$h=\alpha \cdot \left[581W^{0.541}(1-0.0075T_{w})\right]$ [31]
	Foot roll section (W)	1.09	126.00	
	II	2.02	76.40	$h=\;\beta \cdot \left[0.35+0.13W\right]$ [5]
	III	3.52	75.10	
	IV	5.08	74.70	
	V	7.13	34.80	
	VI	9.19	27.70	
	VII	13.53	29.30	
	VIII	18.08	22.50	
	IX	20.37	9.20	
	X	25.51	18.60	
Air Cooling Zone	/		$q=\varepsilon \sigma ({T_{b}}^{4}-{T_{amb}}^{4})$	
			$\varepsilon =0.85/[1+exp{\left(42.68-0.02682T_{b}\right)}^{0.0115}]$ [31]	

**Table 2:** Nucleation parameters in the CAFÉ model.

Nucleation Parameter	Δ*T_S,max_* [K]	Δ*T_S,σ_* [K]	*n_S_*	Δ*T_V,max_* [K]	Δ*T_V,σ_* [K]	*n_V_*
Value	1	0.1	1 × 10^8^	4.3	1.1	3 × 10^10^

**Table 3:** Kinetic parameters of dendrite growth in the CAFÉ model.

Composition	*c*_0_ [wt.%]	*M* [K·(wt.%)^−1^]	*K*_0_	*D* [m^2^·s^−1^]	*Γ* [m·K]
C	0.163	−80.61	0.16	1.10 × 10^−8^	3 × 10^−7^
Si	0.0286	−16.64	0.55	8.50 × 10^−9^	
Mn	0.525	−5.20	0.71	2.40 × 10^−9^	
P	0.021	−28.65	0.26	4.60 × 10^−9^	
S	0.0042	−38.23	0.03	3.50 × 10^−9^	
Cr	0.021	−1.63	0.91	3.30 × 10^−9^	
Al	0.044	4.74	1.17	2.47 × 10^−8^	
Ti	0.016	−13.86	0.28	4.4 × 10^−9^	

**Table 4:** *T_L_*, *T_S_*, and *α_3_* of steel with different C, Mn, and Si contents.

Element	Content [wt.%]	*T_L_* [K]	*Ts* [K]	*α*_3_ [m·s^−1^·K^−3^]	Content [wt.%]	*T_L_* [K]	*Ts* [K]	*α*_3_ [m·s^−1^·K^−3^]
C	0.13	1514	1474	1.221 × 10^−5^	0.17	1510	1467	8.396 × 10^−6^
	0.14	1513	1473	1.105 × 10^−5^	0.18	1509	1465	7.703 × 10^−6^
	0.15	1511	1471	1.004 × 10^−5^	0.19	1509	1464	7.129 × 10^−6^
	0.16	1511	1469	9.161 × 10^−6^	0.20	1508	1461	6.599 × 10^−6^
Mn	1.25	1512	1470	9.735 × 10^−6^	1.45	1511	1469	9.135 × 10^−6^
	1.30	1512	1469	9.580 × 10^−6^	1.50	1511	1469	8.992 × 10^−6^
	1.35	1512	1470	9.427 × 10^−6^	1.55	1511	1468	8.852 × 10^−6^
	1.40	1512	1469	9.280 × 10^−6^	1.60	1510	1468	8.715 × 10^−6^
Si	0.20	1512	1471	9.954 × 10^−6^	0.40	1509	1465	7.791 × 10^−6^
	0.25	1511	1469	9.332 × 10^−6^	0.45	1508	1463	7.365 × 10^−6^
	0.30	1511	1468	8.770 × 10^−6^	0.50	1507	1462	6.974 × 10^−6^
	0.35	1511	1466	8.257× 10^−6^	0.55	1506	1460	6.615× 10^−6^

**Table 5:** Standard error and confidence intervals after fitting.

Number	Value	Standard Error	99% Confidence Interval	R^2^ (COD)
a	0.96819	0.03102	(0.90544, 1.03095)	0.98670
b	0.98189	0.04838	(0.88403, 1.07975)	
c	1.67767	0.13735	(1.39985, 1.95549)	
d	0.97364	0.05069	(0.87111, 1.07617)	
e	0.98738	0.05275	(0.88068, 1.09408)	
f	0.96392	0.04055	(0.88189, 1.04595)	
g	−678.9288	18.41823	(−716.1832, −641.67441)	

**Table 6:** Industrial test schemes.

Schemes	Casting Speed [m·min^−1^]	S-EMS Current [A]	S-EMS Frequency [Hz]
Scheme 1	0.45	0	0
Scheme 2	0.45	570	5
Scheme 3	0.42	570	5
Scheme 4	0.42	570	3.5
Scheme 5	0.42	420	3.5

## Data Availability

The original contributions presented in the study are included in the article; further inquiries can be directed to the corresponding author.

## Nomenclature

The following abbreviations are used in this manuscript:

***f_S_***	solid fraction	**Δ*****T_v,max_***	volume nucleation undercooling
**C**	carbon	**Δ*****T_v,σ_***	standard deviation of the volume during undercooling
**Mn**	manganese	***n_v_***	volume nucleation density
**Si**	silicon	***c*****_0_**	composition of molten steel
***T***	local temperature, K	***k***	equilibrium solute partition coefficient
***l***	thermal conductivity, W·m^−1^·K^−1^	***G***	Gibbs–Thompson coefficient
***ρ***	density, kg·m^−3^	***M***	liquidus slope
***H***	enthalpy, kJ·kg^−1^	***D***	solute diffusion coefficient
***L_P_***	latent heat, J·kg^−1^	***M_max_***	maximum value
***c_p_***	specific heat, J·kg^−1^·K^−1^	***M_min_***	minimum value
***T*****_0_**	initial temperature, K	***G***	temperature gradient at the dendrite tip, K·m^−1^
***T*****_1_**	casting temperature, K	***N*****_0_**	heterogeneous nucleation density, m^−3^
***q_m_***	heat flux of the mold, kW·m^−2^	**Δ*****T_N_***	heterogeneous nucleation undercooling, K
***B***	coefficient related to mold cooling conditions, kW·m^−2^·s^−1/2^	**Δ*****T_C_***	undercooling at the dendrite tip, K
***t***	residence time of molten steel in the mold, s	***V***	dendrite tip growth rate, m·s^−1^
***q_a_***	average heat flux, kW·m^−2^	**Δ*****T*****_0_**	temperature difference between the equilibrium liquid phases, ΔT_0_ = c_0_M(k − 1)/k
***l***	length of the mold, m	***a*****_1_**	constant associated with the alloy system
***V***	casting speed, m·s^−1^	***L***	cooling rate, K·s^−1^
**Δ*****T_n_***	mean undercooling, K	***y***	distance from the CET beginning position to the slab surface, mm
**Δ*****T_σ_***	standard deviation of undercooling, K	***x*****_1_**	superheat, K
***N_max_***	maximum nucleation density, m^−3^	***x*****_2_**	specific water flow, L·kg^−1^
**Δ*****T***	undercooling, K	***x*****_3_**	actual casting speed, m·min^−1^
**Δ*****T_s,max_***	surface nucleation undercooling	***x*****_4_**	C content, wt.%
**Δ*****T_s,σ_***	standard deviation of the surface during undercooling	***x*****_5_**	Mn content, wt.%
***n_s_***	surface nucleation density	***x*****_6_**	Si content, wt.%.
